# Post-traumatic stress disorder and its associated factors among internally displaced people due to conflict in Northwest Ethiopia

**DOI:** 10.3389/fpubh.2025.1386566

**Published:** 2025-03-10

**Authors:** Mamaru Melkam, Girmaw Medfu Takelle, Getasew Kibralew, Girum Nakie

**Affiliations:** Department of Psychiatry, University of Gondar College of Medicine and Health Science, Gondar, Ethiopia

**Keywords:** Ethiopia, internally displaced people, post-traumatic stress disorder, associated, factor

## Abstract

**Background:**

Post-traumatic stress disorder (PTSD) is a mental health condition that develops after being exposed to trauma, such as experiencing or witnessing life-threatening events, including war and other natural disasters. Despite the high levels of conflict, little attention has been given to post-traumatic stress disorder, particularly in low- and middle-income countries. Therefore, this study aimed to determine the prevalence of post-traumatic stress disorder and its associated factors among internally displaced people in Northwest Ethiopia.

**Methods:**

A cross-sectional study design was employed from June to July 2022 in Northwest Ethiopia among internally displaced people. Simple random sampling was used to recruit 410 study participants. The standard tools used in this study included the Post-Traumatic Stress Disorder Checklist for DSM-5 (PLC-5), Kessler-10, and Oslo Social Support Scale to assess various variables. Binary logistic regression analysis was used to identify factors associated with post-traumatic stress disorder. Statistically significant factors were selected at a 95% confidence interval (CI) with adjusted odds ratio (AOR).

**Results:**

The prevalence of post-traumatic stress disorder among internally displaced people was 54.3%, with a 95% CI (49.5, 59.3). Current substance use [AOR 95% CI: 2.01(1.16, 3.48)]; living arrangements, such as living alone or with non-relatives [AOR = 2.13; 95% CI (1.17, 3.86) and AOR = 2.39; 95% CI: (1.21, 4. 70), respectively]; being violated [AOR = 2.49; 95% CI: (1.26, 4.94)]; and psychological distress [AOR = 3.21; 95% CI: (4.35, 9.34)] were significantly associated with post-traumatic stress disorder.

**Conclusion:**

The prevalence of post-traumatic stress disorder among internally displaced people was high. Therefore, stakeholders should provide immediate interventions that include further assessments using diagnostic criteria. In addition, Eye Movement Desensitization and Reprocessing psychotherapy along with selective serotonin reuptake inhibitors as psycho-pharmacological treatment are recommended. Furthermore, efforts should be made to reduce the identified risk factors to improve outcomes for individuals with post-traumatic stress disorder.

## Introduction

Internally displaced people (IDP) are groups of individuals who have been displaced from their original places of residence or homes but have resettled within the borders of their own territory or country (1). They are often displaced due to circumstances similar to those faced by refugees, such as conflicts between regions within the country or organized warfare. However, unlike refugees, IDP do not cross international borders (2). According to the fifth edition of the *Diagnostic and Statistical Manual of Mental Disorders* (DSM-5), post-traumatic stress disorder (PTSD) is characterized by symptoms such as intrusive thoughts, avoidance behaviors, changes in mood and cognition, hyperarousal, and alterations in arousal and reactivity (3). These symptoms often arise as a result of emotional trauma and exposure to multiple psychosocial stressors (4). Witnessing traumatic events or directly experiencing situations such as murder, threats, kidnapping, the death of loved ones or friends, loss of home, or malnutrition can lead to PTSD (5). Early identification of the cause and a commitment to treating PTSD in individuals exposed to trauma are crucial for a better prognosis (6). The majority of displaced people are repeatedly exposed to various stressors and traumatic events, both prior to their journey and during travel to a safer location (7, 8).

Global instability and conflicts contribute to PTSD in more than 20 sub-Saharan African countries (9). Among the world’s displaced populations, the majority is found in low- and middle-income countries, accounting for more than 26% of the total (10, 11). Displaced individuals often endure numerous traumatic experiences that vary in their impact on morbidity and mortality. These experiences frequently lead to mental health issues, many of which have long-lasting effects. Among the various mental disorders, post-traumatic stress disorder (PTSD) is one of the most common conditions following exposure to trauma (12). PTSD is also one of the most significant psychological disorders that arise after exposure to traumatic or stressful events, which often occur at various times (12). According to the DSM-5, a traumatic event is defined as exposure to actual or threatened death, serious injury, or sexual violation. Such exposure can lead to symptoms such as re-experiencing, avoidance, negative alterations in cognition and mood, and heightened arousal (13). However, the ICD-10 diagnostic criteria only focus on events that cause significant distress to individuals (14). Several studies have shown that individuals who remain in their own country after displacement often experience worse mental health outcomes (15, 16). The majority of war-displaced people experienced a range of traumatic events, including exposure to death, physical injury, and emotional or sexual trauma (17). Based on psychological and cognitive theories, post-traumatic stress disorder is officially recognized as one of the most significant issues related to trauma, requiring urgent attention and emphasis (18). According to the World Health Organization (WHO), stress-related diseases and mental illness were projected to become the second leading cause of disability by the year 2020 (19). Approximately 4% of the global population is affected by post-traumatic stress disorder, making it a major contributor to the worldwide disease burden (20). According to a systematic review, the prevalence of PTSD among IDP ranged from 3 to 88% (21). The pooled prevalence of PTSD among displaced populations and refugees in Africa was found to be 56.35% (22). A meta-analysis conducted involving the Iranian population following disasters and wars showed that the pooled prevalence of PTSD was 47% (23). The prevalence of PTSD, as indicated by a meta-analysis study, ranged from 4.10 to 67.07% in China and was 33.5% in Turkey (17, 24). A 6-month study in Nigeria that compared populations exposed to conflict with those not exposed revealed that the prevalence rates of PTSD were 60 and 14.5%, respectively (25). The burden of PTSD among displaced people in Sudan and Uganda was 25 and 76%, respectively (26, 27). Other studies conducted in Ethiopia reported prevalence rates of 15.4, 37.3, and 59.8% (11, 16, 28).

According to the PLC-V, PTSD symptoms are described as one of the most prevalent mental health conditions among IDP. Factors such as low educational status, unemployment, older age, low economic status, and being unmarried have been identified as significant associated factors (29–31). In addition, factors such as being female, having a low educational level, existing medical conditions, a family history of mental illness, and a history of previous mental illness have also been associated with post-traumatic stress disorder (16, 24, 32). Direct exposure to trauma and psychological distress have also been linked to post-traumatic stress disorder (11). Substance use and poor social support have been identified as associated factors in various studies (33–35). Numerous studies have indicated that factors such as being violated, being displaced multiple times, destruction of personal property, and directly witnessing the murder of a family member are significantly associated with PTSD among IDP (11, 16, 36–39).

In African countries, particularly in sub-Saharan Africa, the majority of people with PTSD do not receive treatment, exposing them to an increased risk of developing chronic symptoms (9, 11). The burden of post-traumatic stress disorders in Ethiopia has not been studied as much as the internal conflicts within ethnic groups, including the war that lasted for over a year and displaced many people from their original homes. Therefore, this study assessed the prevalence of post-traumatic stress disorder and its associated factors among internally displaced people in Northwest Ethiopia.

## Methods

### Study design and period

A community-based cross-sectional study design was used from June to July 2022.

### Study area

Gonder and Debark are towns located in the Amhara region of Northwest Ethiopia. Gondar is 700 km from Addis Ababa, which is the capital city of Ethiopia, and 172 km from Bahir Dar, the capital of the Amhara region. Debark is situated 830 km from Addis Ababa and 106 km from Gondar. Currently, there are internally displaced people from various areas due to conflicts within the Amhara and Tigray regions. The majority of the displaced people come from various areas, especially from the Amhara region (on the border with Tigray) and the Tigray region. The IDP are located in Gondar and Debark in the Amhara region, which serve as formal camps for the displaced individuals. The total number of displaced people aged 18 and over in both IDP centers is 2,757, of whom 725 are men and 2,032 are women. A total of 1,470 people are from the Gondar IDP center, and 1,287 are from the Debark IDP camp. The displaced people have been staying in the IDP centers for varying periods of time.

### Study population and sampling technique

The study population included all displaced people residing in the Gondar and Debark IDP centers. Specifically, the study population consisted of all people living in the temporary camps in both Gondar and Debark IDP centers during the data collection period.

A simple random sampling technique was employed to select participants. The list of residents was obtained from the leaders/coordinators of the temporary camps, and participants were selected using a computer-generated random method. A total of 219 and 191 study participants were proportionally allocated to the Gondar and Debark IDP camps, respectively. For eligible participants who were absent from their assigned camps, the interviewer revisited the camp/area at another time during the data collection period.

#### Eligibility criteria

All individuals living in the IDP centers and aged 18 and over were included in this study. Individuals who were seriously ill at the time of data collection were excluded.

### Sample size and sampling procedure

The sample size (n) was determined using the single population proportion formula with the following parameters: a margin of error of 5%, a 95% confidence interval, and a *p*-value of 58.4%, derived from a previous study conducted in Ethiopia (16), and a 10% non-response rate (40).


$$n=\frac{Z@/2\;p\left(1-p\right)}{d^{2}}$$

Where d is the margin of error (5%) in the case of our study (41).

The sample size n is calculated using the following formula:


$$n=\frac{Z@/2\;p\left(1-p\right)}{d^{2}}$$

Substituting the values,


$$n=\frac{{\left(1.96\right)}^{2}X\;\left(0.584\times 0.416\right)}{{\left(0.05\right)}^{2}}$$

n=373

After adding a 10% non-response rate to the calculated sample size of 373, the total sample size was adjusted to 410 residents at the IDP centers. The double population proportion formula was used to calculate the sample size based on associated factors, but the resulting sample size was smaller than that calculated using the outcome variables. Therefore, the final sample size was set at 410.

### Study variables

#### Dependent variables

PTSD symptoms were considered dependent variables.

#### Independent variables

Sociodemographic factors including sex, age, marital status, ethnicity, religion, education, occupation, length of time since leaving their home, types of traumatic events experienced, frequency of displacements, psychological distress, substance use, social support, and being violated were considered independent variables.

### Data collection measurement tool

#### Demographic data

This provided baseline information about the participants’ profiles, including sociodemographic and IDP-related factors such as sex, age, ethnicity, religion, education, occupation, marital status, number of times displaced from their homes, types of traumatic events experienced, and frequency of displacements.

#### Post-Traumatic Stress Disorder Checklist for DSM-5 (PCL-5)

The PCL-V is a standardized self-reporting rating scale used to assess the 20 DSM-V symptoms of PTSD (42). A total score is calculated by summing the 20 items, with possible scores ranging from 0 to 80. The items are rated using a 5-point Likert scale (0 = Not at all, 1 = A little bit, 2 = Moderately, 3 = Quite a bit, and 4 = Extremely), with a cutoff point of ≥33 (42).

#### Psychological distress assessment using the Kessler-10 scale

Each item is scored on a scale from 1 (‘none of the time’) to 5 (‘all of the time’). The scores of the 10 items are summed, yielding a minimum score of 10 and a maximum score of 50. Lower scores indicate low levels of psychological distress, while higher scores indicate high levels of psychological distress (43).

#### Use of substance

Current substance use: it is defined as the use of one or more substances (alcohol, khat, and/or cigarettes) within the last 3 months.

Lifetime substance use: this is defined as the use of one or more substances (alcohol, khat, and/or cigarettes) at any point in one’s lifetime.

#### Oslo Social Support Scale

This questionnaire is used to assess social support status and is a validated tool in the study area. The Oslo Social Support Scale has a total score ranging from 3 to 14, with three broad categories: poor social support 3–8, moderate social support 9–11, and strong social support 12–14 (44).

#### Being violated

Yes-or-no questions were used to determine whether the individuals experienced violation during the conflict. For example: “Have you experienced physical, sexual, emotional, and psychological harm due to the event?”

### Data collection procedure

Data were collected using face-to-face, interviewer-administered questionnaires. The data collection was conducted by four BSc psychiatry nurses, with regular supervision by two psychiatry professionals. The interviews were conducted in private, following a computer-generated method. The confidentiality of the data and the privacy of the participants were maintained. The questionnaires did not include the respondents’ names, and the interviews were conducted in the absence of a third party to ensure that no one else could overhear the responses.

### Data quality control

The questionnaire was initially designed in English, translated into Amharic, and then translated back to English by language experts and mental health specialists to ensure consistency. Before the actual data collection, a pretest was carried out among 5% of the overall sample size at the Dabat IDP center, which was not part of the primary data collection site. The Cronbach’s alpha results for the pre-test were 0.83 for the outcome variable measurement tool PCL-V, 0.77 for the Kessler-10 scale, and 0.69 for the Oslo Social Support Scale. The data were collected by four BSc psychiatry professionals and regularly supervised by two additional psychiatry professionals. The data collectors were trained for 1 day on how to obtain written informed consent and conduct interviews with the study participants. Humanitarian responder teams, including psychiatrists, were available to assist the study participants who required additional help after the interview. These participants were referred for further assessments and interventions.

### Data processing and analysis

The data were coded and entered using Epi-data version 4.6.0. These data were then exported and analyzed using SPSS version 20. Descriptive statistics, such as means, medians, frequencies, percentages, and standard deviations, were calculated from the data. A 95% confidence interval and adjusted odds ratios were calculated using logistic regression analysis to account for confounding variables. To determine whether there was a significant correlation, variables from the bivariable logistic regression analysis with a *p*-value of less than 0.2 were included in the multivariable logistic regression analysis. Outcomes and independent variables were included in both multivariable logistic regression and bi-variable logistic regression analyses, one at a time, to find associations and assess their presence and strength. Statistical significance of the related factors was established at a *p*-value of 0.05. The model’s goodness-of-fit was assessed using the Hosmer–Lemshow test (68.9%). The variance inflation factor (VIF) was <1.06, and the tolerance rate of it was <10, which are acceptable values for checking multicollinearity (45).

## Results

### Sociodemographic characteristics of the study participants

A total of 398 participants were included in this study, with a response rate of 97.1%. Of the participants, 82.4% were women. The mean age of the study participants was 37 years, with a standard deviation of ±13.13 and an age range of 18–78 years. More than half of the participants (71.1%) were Orthodox religious followers, and 74.1% were from the Amhara region. Among the respondents, 36.4% had no formal education, 36.7% lived alone, and 30.7% were farmers (Table 1).

**Table 1:** Sociodemographic factors of the study participants.

Variables	Category	Frequency	Percentage
Sex	Male	70	17.6%
	Female	328	82.4%
Age	18–40	270	67.8%
	41–60	103	25.9%
	>61	24	6%
Religion	Orthodox	283	71.1%
	Muslim	92	23.1%
	Protestant and others	23	5.8%
Marital status	Single	104	26.1%
	Married	193	48.5%
	Divorced	71	17.8%
	Widow	30	7.5%
Ethnicity	Amhara	295	74.1%
	Oromo	23	5.8%
	Tigray and others	80	20.1%
Educational status	Uneducated	145	36.4%
	1–8 grade	132	33.2%
	9–12 grade	97	24.4%
	Diploma	24	6.0%
Occupation	Farmer	122	30.7%
	Merchant	144	36.2%
	Student	54	13.6%
	Housewife	78	19.6%
Living arrangements	Alone	146	36.7%
	With parents	108	27.1%
	With relatives	82	20.6%
	Others*	62	15.6%

^*^Others refers to those living with friends or non-parents.

### Trauma-related factors of the study participants

Among the participants, 51.5% were displaced during wartime and more than half (51.5%) witnessed the destruction of personal property. In addition, 61.1% were displaced for more than 12 months. More than half (51.3%) experienced displacement once in their lifetime, and 54.8% witnessed the war. The majority of the study respondents (85.7%) faced a lack of food or water in the camps (Table 2).

**Table 2:** Trauma-related factors among internally displaced people in Northwest Ethiopia.

Variables	Category	Frequency	Prevalence
The time of escape from their homes	Before the war	48	12.1%
	During the war	205	51.5%
	After the war	145	36.4%
Types of traumatic events	Physical assault	36	9.0%
	Being in a war-fighting situation	139	34.9%
	Destruction of personal property	205	51.5%
	No access to medical care during the event	18	4.5%
Duration since displacement	< 9 months	32	8.0%
	9–12 months	123	30.9%
	>12 months	243	61.1%
Frequency of displacement	One time	204	51.3%
	Two times	129	32.4%
	Three times or more	65	16.3%
Types of problems in the camp	Lack of housing or shelter in the camp	57	14.3%
	Lack of food or water in the camp	341	85.7%
Types of exposure that makes you terrified	Direct	89	22.4%
	Witnessed	218	54.8%
	Family or close friend injury	88	22.1%
	Job-related exposure	3	0.8%

### Psychosocial and substance use factors

Among the study participants, more than half (64.8%) had psychological distress. In addition, 26.1% reported current substance use. The majority of the respondents (78.1%) had poor social support, while 15.1% had moderate social support and 6.8% had strong social support (Table 3).

**Table 3:** Psychosocial, substance use, and clinical factors among IDP in Northwest Ethiopia.

Variables	Category	Frequency	Percentage
Psychological distress	Yes	258	64.8%
	No	140	35.2%
Substance use	Yes	104	26.1%
	No	294	73.9%
Social support	Poor social support	311	78.1%
	Moderate social support	60	15.1%
	Strong social support	27	6.8%

### Prevalence of post-traumatic stress disorder among the IDP

**Figure 1:**
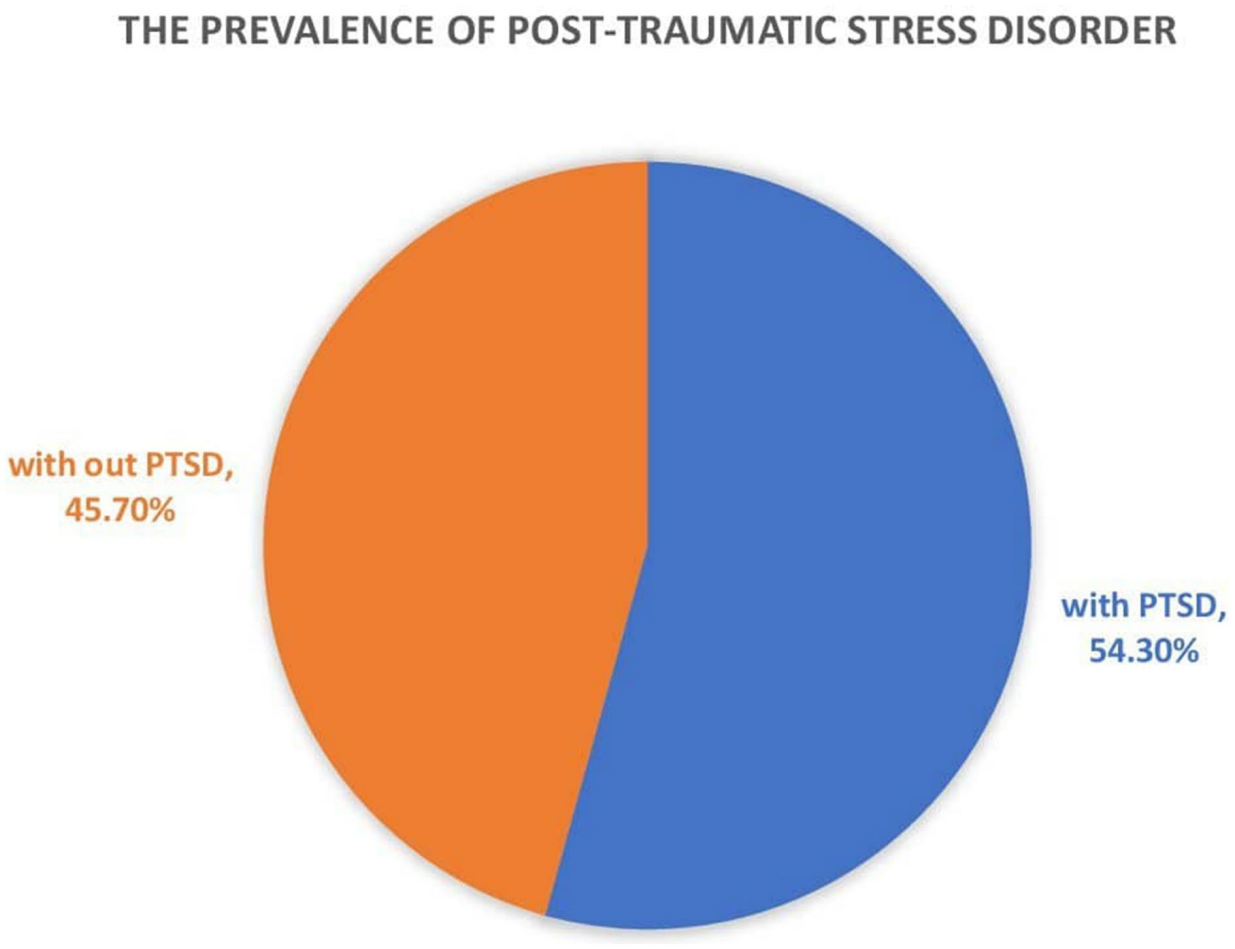
Prevalence of post-traumatic stress disorder among internally displaced people in Northwest Ethiopia.

The prevalence of post-traumatic stress disorder among the internally displaced people was 54.3%, with a 95% CI (49.5, 59.3) (Figure 1).

### Associated factors of post-traumatic stress disorder

In the bivariable logistic regressions analysis, educational status, living arrangements, time of escape from their homes, types of problems faced during the war, being violated, current substance use, poor social support, and psychological distress were all associated with post-traumatic stress disorder, with a *p*-value of less than 0.2. These variables were then included in multivariable logistic regression analysis. In both bivariate and multivariable logistic regression analyses, substance use, living arrangements, being violated, and psychological distress were statistically significantly associated with post-traumatic stress disorder.

Substance use was 2.01 times more frequent in individuals with PTSD diagnosis compared to those who had not used substances [AOR = 95% CI: (1.16, 3.48)]. The likelihood of developing PTSD was 2.13 and 2.39 times higher among those living alone and living with non-relatives, respectively, as compared to those living with parents or families [AOR = 95% CI (1.17, 3.86)] and [AOR = 95% CI: (1.21, 4. 70)]. Being violated was 2.49 times more likely to result in PTSD compared to not being violated [AOR = 95% CI: (1.26, 4.94)]. The risk of PTSD was 3.21 times higher among those with psychological distress compared to those without psychological distress [AOR = 95% CI: (4.35, 9.34)] (Table 4).

**Table 4:** Bi-variable and multivariable regression analyses of the factors associated with PTSD among IDP.

Variables	Category	PTSD		COR	AOR
		Yes	No		
Educational status	Uneducated	75	70	1	1
	Grade 1–8	66	66	0.93 (0.58, 1.49)	0.67 (0.38, 1.19)
	High school	59	38	0.45 (0.86, 2.44)	0.98 (0.52, 1.88)
	Diploma and above	16	8	1.88 (0.75, 0.63)	1.03 (0.33, 3.25)
Living arrangements	With parents	64	82	1	1
	Alone	68	40	2.17 (1.30, 3.62)	**2.13 (1.17, 3.86)**
	With non-relatives	50	29	2.34 (1.34, 4.09)	**2.39 (1.21, 4. 70)**
	With relatives	31	31	1.28 (0.70, 2.32)	1. 64 (0.81, 3.31)
Current substance use	Yes	83	33	2.62 (1.65, 4.14)	**2.01 (1.16, 3.48)**
	No	133	147	1	1
Time of escape	Before the war	21	28	1	1
	During the war	107	98	1.40 (0.75, 2.64)	1.41 (0.67, 2.98)
	After the war	88	57	1.98 (1.02, 3.84)	1.69 (0.77, 3.71)
Poor social support	Poor	160	153	0.62 (0.27, 5.68)	0.70 (0.25, 2.00)
	Moderate	39	23	1.09 (0.42, 2.80)	1.03 (0.32, 3.32)
	Strong	17	10	1	1
Being violated	Yes	53	17	3.15 (1.75, 5.68)	**2.49 (1.26, 4.94)**
	No	163	165	1	1
Types of problems	Lack of housing	36	21	1	1
	Lack of food and water	180	161	0.65 (0.36,1.16)	0.52 (0.25,1.07)
Psychological distress	Yes	35	77	0.14 (0.09, 0.22)	**3.21 (4.35, 9.34)**
	No	181	105	1	1

Bold values indicates significantly associated.

## Discussion

The major objective of this research was to determine the prevalence of post-traumatic stress disorder and its associated factors among internally displaced people in Northwest Ethiopia. The prevalence of post-traumatic stress disorder among the internally displaced people was 54.3%, with a 95% CI (49.5, 59.3). In low- and middle-income countries, post-traumatic stress disorder is becoming a significant public health issue due to exposure to various human-made disasters, such as ethnic conflicts, terrorist attacks, and civil wars. In Ethiopia, many people have been displaced due to ethnic conflicts and are living in temporary camps. This finding is in line with that of another study conducted in South Ethiopia, which reported a prevalence of 58.4% (16).

The results are lower than those of other studies conducted in Mai Kadra (59.8%) (11), Kenya (82%) (46), Nigeria (74%) (47), Iraq (60%) (48), and Uganda (67%) (27). The probable reason for this discrepancy might be the influence of factors such as witnessing the events or learning about traumatic events from family members, as seen in the Nigeria study, which were not included in our study. Another reason for the discrepancy could be the severity of the trauma in the Mai Kadra massacre, with people witnessing many others' deaths in a short timeframe.

In addition, these findings are higher than those of other studies conducted in Northwestern Nigeria (42.2%) (49), Turkey (21.4%) (50), and Iraq (38%) (51). This discrepancy could be due to differences in the measurement tools used across countries, which may affect the overall prevalence of PTSD. Some of the previous studies were carried out using PTSD checklists, which might explain the discrepancy. This suggests that checklists could be stronger than screening measurement tools, potentially leading to a higher reported burden of PTSD than screening measurement tools, potentially leading to a higher reported burden of PTSD. Living alone and living with a non-relative were statistically significantly associated with post-traumatic stress disorder. This finding is consistent with those of other studies carried out in different countries, including Ethiopia (28, 52). This association might be due to the fact that individuals who experience traumatic crises alone may struggle to establish effective coping mechanisms after the trauma, while those with strong family support during traumatic exposure may be better able to cope (52). The absence of assistance for those who are physically unable to care for themselves, along with a lack of emotional support and resources for advice, could increase the likelihood of developing PTSD (28). Another possible reason for the association could be poor family support, particularly among individuals who experienced psychosocial adjustment problems while living alone. The absence of close family members or parents could increase the risk of developing post-traumatic stress disorder, as emotional support is essential for coping. The other factor associated with post-traumatic stress disorder was the current use of substances. This finding is consistent with those of other studies carried out in different countries. A possible reason for the association could be that individuals use substances to alleviate traumatic pain and emotional distress (53, 54). Another possibility is that substance use temporarily reduces flashbacks and the reliving of traumatic experiences in people with PTSD, as well as help them cope with other stressful events in their lives (55).

Being violated was also associated with post-traumatic stress disorder. Due to cultural fears of embarrassment, rejection by their husbands, or retaliation by their assailants, victims of rape often do not report the crime that can eventually lead to PTSD (56). A possible reason for the association between violation and PTSD is the emotional distress and embarrassment caused by others, which can contribute to the development of post-traumatic stress disorder.

Psychological distress was also identified as one of the factors associated with PTSD among the internally displaced people. This result is consistent with those of other studies (16, 49). The presence of other mental illnesses, such as psychological distress, depression, or anxiety, may be due to the fact that psychologically distressed individuals are more vulnerable to developing PTSD (16). Comorbid mental illness may make it difficult for a person to fully process the effects of the traumatic experience and the associated emotions, which can hinder their ability to heal (57). Another reason for this association could be that having other psychiatric problems increases the likelihood of developing PTSD and other mental illnesses, which, in turn, leads to a poorer quality of life and impaired functional status (57). Another explanation is that individuals with a history of brain injury—one of the risks for many mental illnesses—may be more likely to develop PTSD than those without such a history.

### Practical implications for public health

1. Stakeholders should initiate conflict resolution and management strategies to minimize and/or prevent instability caused by ethnic, tribal, or political conflicts. Mental health issues contribute significantly to all-cause morbidity and mortality rates globally. This study provides valuable evidence for policymakers regarding PTSD among internally displaced people.
2. Study participants need therapeutic interventions, either pharmacological or psychotherapeutic, for their PTSD, following reassessment based on diagnostic criteria. Specifically, providing Eye Movement Desensitization and Reprocessing psychotherapy and selective serotonin reuptake inhibitors as a psycho-pharmacological intervention may be more effective.
3. Future researchers are encouraged to use advanced methodologies to identify the exact causes of PTSD, which can enhance the effectiveness of interventions. A mixed-methods approach, combining qualitative and quantitative studies, is recommended to capture more in-depth traumatic narratives, rather than relying solely on direct questionnaires, such as those used in focus group discussions.

### Strengths and limitations

The strength of this research lies in the use of a standardized tool to screen for PTSD. The data were collected through interview-administered assessments of PTSD in a timely manner. One limitation of the study is its cross-sectional design, as it cannot establish cause-and-effect relationships. In addition, social desirability bias might have influenced the responses to certain questions, such as those related to substance use.

## Conclusion

The prevalence of post-traumatic stress disorder among the internally displaced people in Northwest Ethiopia was 54.3%. Factors such as substance use, living arrangements, being violated, and psychological distress were statistically significant contributors to post-traumatic stress disorder. Therefore, immediate therapeutic interventions for displaced people are essential, with support from the health bureau and non-governmental organizations. Restrictions on substance use should be implemented, and psychological distress should be regularly evaluated and treated to reduce the burden of PTSD among internally displaced people.

## Operational definitions

Post-traumatic stress disorder (PTSD): PTSD was measured using the Post-Traumatic Stress Disorder Checklist for DSM-5 (PLC-5). The cut-off point for this screening tool is a score of 33 or higher, based on a total of 20 questions with a five-point Likert scale (58, 59).

Psychological distress: psychological distress was measured using the Kessler-10 scale, which consists of 10 items. The cutoff point for this tool is a score of 19 (43).

Social support: Social support was measured using the Oslo Social Support Scale, which categorizes support into three levels: poor, moderate, and strong, with cut-off points of 3–8, 9–11, and 12–14, respectively (44).

Current and lifetime substance use: current substance use was defined as the use of at least one substance in the past 3 months, while lifetime substance use was defined as the use of at least one substance at any point in the individual’s life (60).

## Data Availability

The original contributions presented in the study are included in the article/supplementary material, further inquiries can be directed to the corresponding author.
